# The Reverse Dual Plane: A Novel Technique for Endoscopic Transaxillary Breast Augmentation

**DOI:** 10.1093/asjof/ojae020

**Published:** 2024-04-05

**Authors:** Zumeng Ya, Lin Xiao, Luheng Zhou

## Abstract

**Background:**

Quite a few Asian patients prefer axillary incision for breast augmentation. However, this surgery needs improvement.

**Objectives:**

To introduce a reverse dual-plane technique through a transaxillary approach and compare it with a transaxillary dual-plane approach.

**Methods:**

Eighty-two patients were divided into Group A (*n* = 40) and Group B (*n* = 42). Axillary incision and endoscope were utilized in the 2 groups. Tebbetts’ dual plane was performed in Group A patients. Patients in Group B underwent our reverse dual-plane technique, in which the upper 70% was subfascial and the lower 30% was subpectoral, with the fascia of the external oblique and anterior serratus being elevated together with the pectoral muscle. The Numeric Pain Rating Scale (NPRS) scores were recorded daily for 7 days. Breast shape and softness, in both sitting and supine positions, were assessed by the patients, and complications were compared.

**Results:**

The NPRS scores of Group B patients were significantly lower than those of Group A patients (*P* < .01). The satisfaction rate of shape and softness in the seated position was not significantly different (*P* > .05). However, in the supine position, only 20 patients (50.0%) in Group A and 32 patients (76.2%) in Group B were satisfied with their breast softness (*P* < .01), and the breasts of the others became stiffer. Breast animation deformity (BAD) occurred in 2 patients in Group A and in no patient in Group B (*P* < .01). Other complications were not significantly different.

**Conclusions:**

Compared with Tebbetts’ dual plane, this procedure significantly reduced pain, improved breast softness, and eliminated BAD, without increasing complications.

**Level of Evidence: 4:**

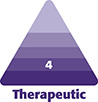

Implant breast augmentation is one of the most frequently performed aesthetic surgical procedures in the world. In the past 2 decades, the popularization of Tebbetts’ dual-plane technique^1,2^ and subfascial plane technique^3^ has resulted in some improvement in alleviating postoperative pain, breast shaping, and decreasing complication incidence.^1-5^ However, patients still complained about significant postoperative pain.^6-12^ Animation deformity, implant palpability, rippling, and implant malposition occurred occasionally.^1,4^ In recent years, some of our patients have been complaining over the phone that their breasts were firming. We asked them to get readmitted to the hospital, after which we found that their breast softness was normal. However, they said that their breasts were still soft when sitting or standing, but they became hard when lying on the back. Therefore, we reexamined their breasts in the supine position, and we found that the supine position did have harder breasts than the seated position. Based on our observations, among patients who did not have this complaint, there were also varying degrees of difference in breast softness between the seated and the supine positions. This dissatisfaction is related to a problem with a negative effect on the BREAST-Q.^13-15^ Unfortunately, the complaint has been neglected by surgeons to such an extent that there are no reports in the literature on this subject. All of these suggest that implant breast augmentation needs further improvement.

In contrast, different from Western countries, quite a few Asians, especially Chinese, dislike inframammary fold (IMF) and areolar incisions, because they do not want to leave a scar in the aesthetic view of their breasts despite recommendations by surgeons.^16-18^ They worry that the symmetric scar on the breasts makes them less attractive. A fair number of patients in Asia prefer axillary incision for breast augmentation. To respect such patient choices, Tebbetts’ dual-plane technique through a transaxillary approach has been performed by many surgeons, especially Asian surgeons, following which there has been a significant improvement in results.^2,16,17^ However, patients have occasionally had some complaints such as postoperative pain, animation deformity, and palpability of implants.^2,4,5,18-20^ The transaxillary endoscopic procedure still leaves plenty of room for improvement. Therefore, in this study, we introduce our novel reverse dual-plane axillary endoscopic breast technique and compare it with the more traditional transaxillary dual-plane technique for achieving patient satisfaction.

## METHODS

A total of 82 patients with a mean age of 35 years (range, 20-52 years) were included in this study from October 2018 to May 2022. The inclusion criteria were primary implantation augmentation, preferably through an axillary incision, with a pinch thickness >1.0 cm. These patients were divided into Tebbetts’ dual-plane group (Group A, *n* = 40) and our modified dual-plane group (Group B, *n* = 42). Randomization was based on the order of operations, with odd numbers assigned to Group A and even numbers to Group B. This study was approved by the ethics committee of Chongqing Vcharm Plastic Surgery Hospital, and all patients had signed consent forms for surgery. All operations utilized axillary incisions with endoscopic assistance, and all were performed under endotracheal intubation and intravenous anesthesia. In Group A patients, the implant pockets were formed as Tebbetts’ Type I and Type II dual planes.^2^ The dissection was subpectoral from the entrance to the new IMF, with the fascia of the anterior serratus and external oblique elevated together with the pectoral muscle. Then, the pectoral muscle was resected at a different level, according to the degree of the breast ptosis (Video 1).

**Figure 1.**
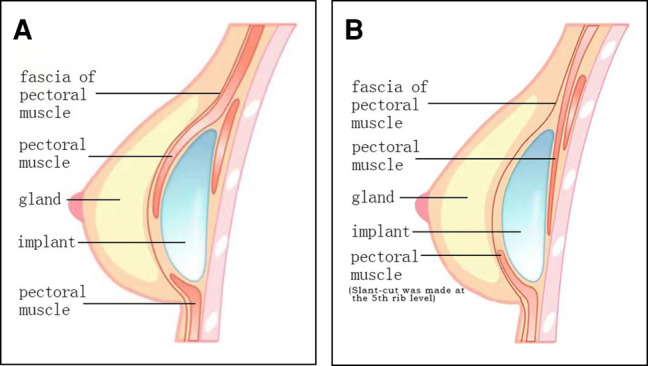
A diagram showing (A) Tebbetts’ dual-plane implant technique and the (B) authors’ modified reverse dual-plane technique.

**Figure 2.**
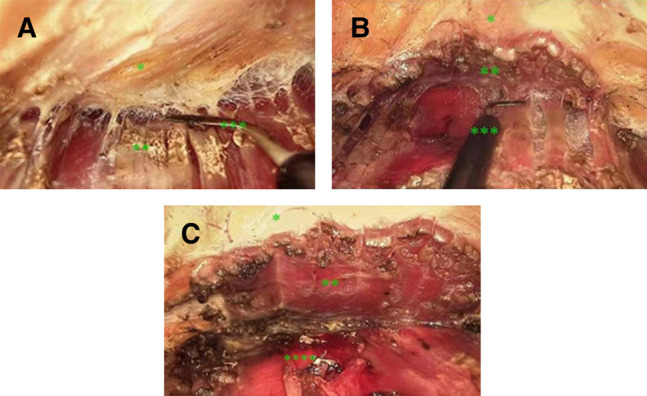
Pocket formation. (A) Subfascial dissection was performed from the entrance to the fifth rib level. (B) Dissection tuned to the submuscular plane with the fascia of the external oblique and anterior serratus elevated. (C) Postdissection. *Fasciae of the pectoral muscle. **The pectoral muscle. ***Electric cutter. ****Rib.

The patients in Group B were operated upon utilizing our reverse dual-plane technique, in which the dissection started beneath the fascia of the pectoral muscle. When the dissection reached the fifth rib, the pectoral muscle was cut in an inclined plane with an electrotome, and then the dissection went gradually deeper under the pectoral muscle, with the fascia of the external oblique and anterior serratus being elevated together. The dissection was completed at the new IMF. If the new IMF was lowered, the dissection was continued under the rectus abdominus muscle fascia. In this manner, a novel dual-plane pocket was formed (Figures 1, 2, Video 2).

Before implant insertion, 2% lidocaine + 0.75% ropivacaine were utilized to block the intercostal nerve in both groups to relieve postoperative pain. We utilized either round or anatomical microtextured implants from Mentor (SILTEX Round Gel Breast Implant, CPG, Mentor Medical Systems BV, Leiden, the Netherlands). Implant insertion sleeves were not utilized. After placement, the upper part of the implant (accounting for ∼70% of its height) was subfascial, and its lower pole was the subpectoral muscle and subfascial of the external oblique and anterior serratus (accounting for ∼30% of the height of the implant) in Group B (Figure 1). Negative pressure drainage was maintained for 24 to 72 h, and cefazolin and levofloxacin were administered intravenously for 2 days for both groups.

From the operative day (POD0) to postoperative Day 7 (POD7), pain scores were recorded daily by the patients utilizing the Numeric Pain Rating Scale (NPRS). On the NPRS, the patients rated their pain quantitatively on a Likert scale from 0 (no pain) to 10 (worst pain). The NPRS scores were compared between Group A and Group B. Statistical analysis was performed utilizing a *t*-test.

All patients were followed up for at least 18 months after surgery (Table 1). During the follow-up period, breast shape and softness were assessed by the patients, who were reminded to compare their breast softness in both the supine and sitting positions. The patients were asked to fill out the satisfaction questionnaires (Table 2) about various outcomes by themselves, including static and dynamic breast shape, softness, surgical scars, effects on physical labor or exercise, and breastfeeding function. At the same time, any complications, including breast animation deformity (BAD), capsular contracture, implant palpability and malposition, and rippling breast, were assessed by the surgeons. Statistical analysis was performed by using the *χ*^2^ test.

**Table 1.** Basic Information About the Patients in Groups A and B

	n	MA (years)	LFUD (months)	MFUD (months)	MVI (cc)
Group A	40	34.5	18	26.5 ± 6.6	272.5
Group B	42	35.3	18	25.9 ± 5.8	275.5

LFUD, least follow-up duration; MA, mean age; MFUD, mean follow-up duration; MVI, mean volume of implants.

**Table 2.** Satisfaction Questionnaire After Implant Breast Augmentation Surgery

Name	Age	Weight	kg	BMI
Date of surgery	Anesthesia method			
Incision	Brand/type/size/of implants		Plane for implant	
Auxiliary means		Surgeon		
Date of follow-up		Follower		
Complications	Hematomas Infection Seroma Capsular contracture Implant displacement or malposition Implant palpability Animation deformity breast Double bubble breast Waterfall breast BIA-ALCL K. Others:			
The above is filled in by the hospital, and the following is filled in by the patient				
Breast size evaluation	Very satisfied Satisfied General Dissatisfied E. Very dissatisfied			
Breast shape evaluation	Very satisfied Satisfied General Dissatisfied E. Very dissatisfied			
Breast softness evaluation in standing/sitting position	Very satisfied Satisfied General Dissatisfied E. Very dissatisfied			
Breast softness in supine position and comparison with sitting position	Very satisfied Satisfied General Dissatisfied Very dissatisfied No difference between the 2 positions Slight hardening compared with the sitting position H. Significant hardening compared with the sitting position			
Nipple sensitivity changes 6 m after surgery	No significant change Slight decrease Significant decrease D. Numb or no sensitivity			
Effects on lactation after surgery	No significant change Slight decrease C. Significant decrease			
Local or systemic discomfort after surgery	No discomfort Significant discomfort Local swelling pain or numbness Breast and adjacent masses Arthragia Fever Other discomforts:			
Spouse comprehensive satisfaction	Very satisfied Satisfied General Dissatisfied Very dissatisfied			
Other satisfaction or dissatisfaction statements)				

## RESULTS

**Figure 3.**
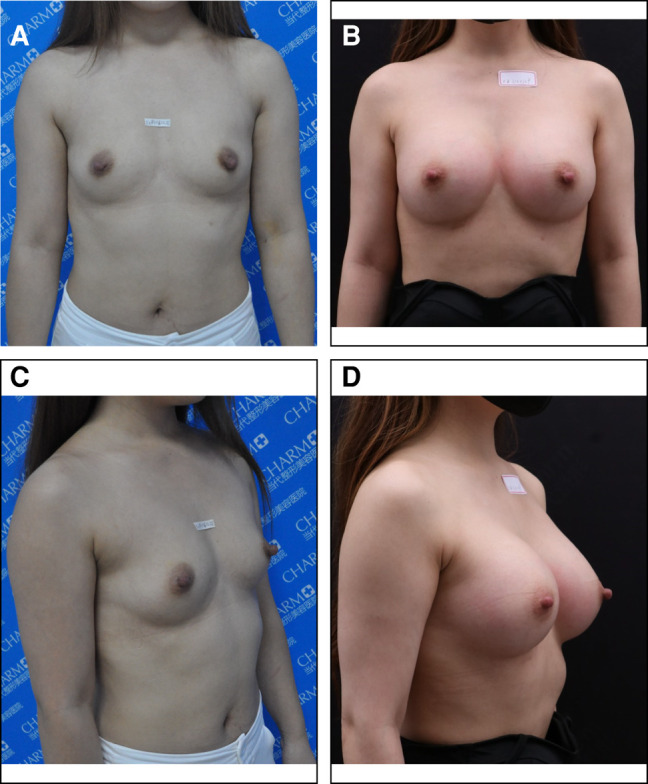
A 31-year-old female presented with a 1.4 cm thickness of soft tissue on the lower quadrant. Through an axillary incision with endoscopic assistance, 335 cc/332 CPG gel implants were inserted in the modified pockets. (A) and (C) show the patient before surgery, and (B) and (D) show the patient 33 months after surgery.

**Figure 4.**
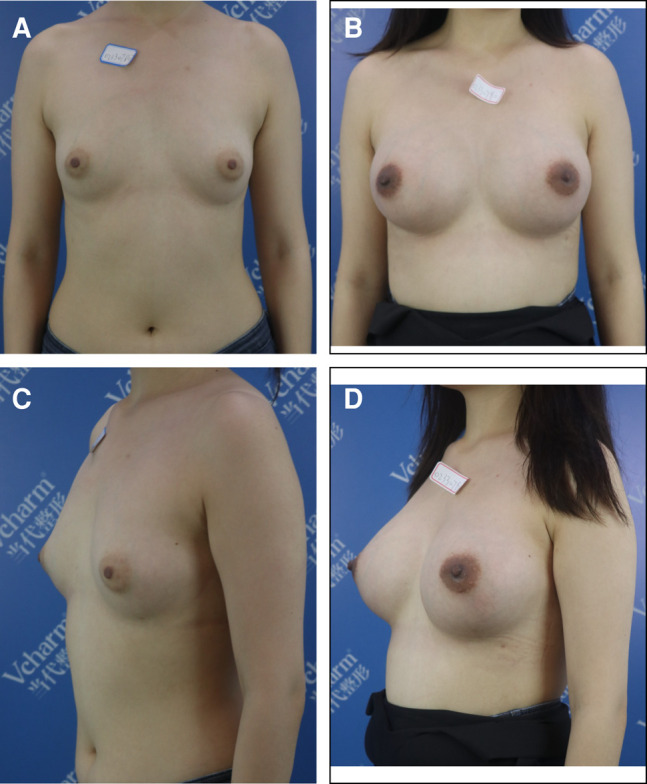
A 35-year-old female presented with a 1.9 cm thickness of soft tissue on the lower quadrant. Through an axillary incision with a modified plane, 280 cc/321 CPG gel implants were utilized. (A) and (C) show the patient before surgery, and (B) and (D) show the patient 24 months after surgery.

**Figure 5.**
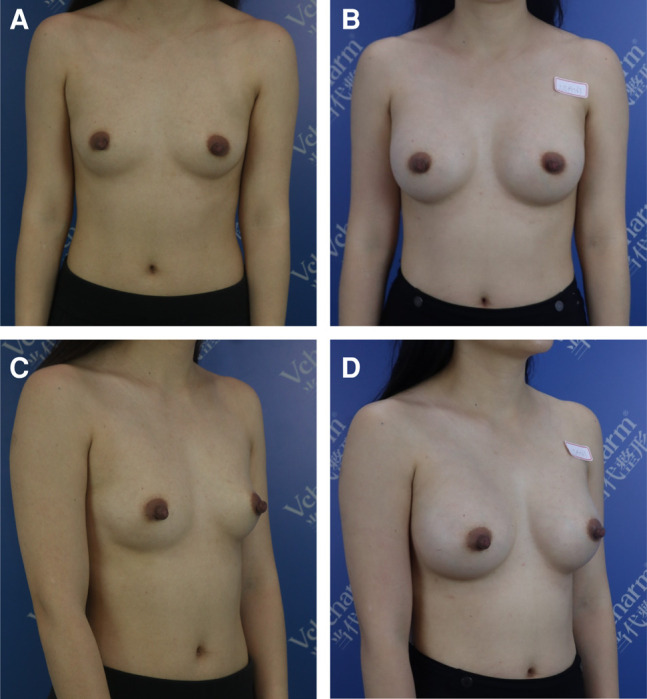
A 37-year-old female presented with a 1.2 cm thickness of soft tissue on the lower quadrant. Through an axillary incision with a modified plane, 255 cc/322 CPG gel implants were utilized. (A) and (C) show the patient before surgery, and (B) and (D) show the patient 19 months after surgery.

Basic information about the patients, including their mean age, mean prosthesis volume, and the average follow-up time (Group A = 26.5 months, Group B = 25.9 months), is provided in Table 1. The breast shape after surgery is shown in Figures 3-5.

### Postoperative Pain

From POD0 to POD7, the NPRS scores of Group B patients were significantly lower than those of Group A patients (Table 3).

**Table 3.** Postoperative the Numeric Pain Rating Scale (NPRS) Scores in Groups A and B

	Group A (*n* = 40)	Group B (*n* = 42)	*P*-value
POD0	8.3 ± 1.6	5.2 ± 0.8	.009*
POD1	7.5 ± 1.2	4.1 ± 0.6	.008*
POD2	7.6 ± 1.0	4.0 ± 0.4	.007*
POD3	6.3 ± 0.9	3.7 ± 0.5	.007*
POD4	5.8 ± 0.7	3.1 ± 0.3	.010*
POD5	5.3 ± 0.7	3.2 ± 0.3	.015*
POD6	4.9 ± 0.6	2.8 ± 0.4	.018*
POD7	4.3 ± 0.8	2.5 ± 0.3	.0016*

POD, postoperative day. *P < .01, its value obtained in a 2-sampled *t*-test.

### Breast Softness in a Different Position

Altogether, 80 patients (80/82, 97.6%) in the 2 groups were satisfied with the softness of their breasts in the sitting or standing position. In Group A, 20 cases (40 sides, 50.0%) of the breasts were significantly harder in the supine position than in the sitting position. However, in Group B, only 10 patients (20 sides, 23.8%) had worse breast softness in the supine position. This difference was significant (*P* < .001; Table 4), which meant that 76.2% of patients in Group B felt no difference in breast softness between the supine and the seated positions.

**Table 4.** Incidence of Breast Softness Changes From the Sitting to Supine Positions in Groups A and B

Group	*n*	Significant difference	Insignificant difference
A	40	20 (40 sides, 50.0%)*	20 (40 sides, 50.0%)*
B	42	10 (20 sides, 23.8%)*	32 (64 sides, 76.2%)*

^*^P < .001, its value obtained in a χ^2^ test.

### Complications After Surgery

There were no hematomas and infections among the patients in the 2 groups. Baker Grade III capsular contracture occurred on 1 side in 1 patient in Group A (her right breast was firm from the 1st month to the 25th month after surgery) and on both sides in 1 patient in Group B (her breasts were firm from the 3rd month after operation). Both these patients required a revision surgery. There were 2 patients with animation deformity in Group A and none in Group B (*P* < .01). Five patients in Group A and 6 in Group B complained of implant palpability (*P* > .05). There was no significant difference between the 2 groups. The postoperative complications are provided in Table 5.

**Table 5.** Incidence of Complications in Groups A and B

Group	*n* (case)	CC (case)	BAD (case)	RB (case)	IP (case)
A	40	1* (2.5%)	2** (5.0%)	1* (2.5%)	5* (12.5%)
B	42	1* (2.4%)	0** (0%)	1* (2.5%)	6* (14.3%)

BAD, breast animation deformity; CC, capsular contracture; IP, implant palpability; RB, rippling breast. *P > .05, **P < .01, its value obtained in a χ^2^ test.

## DISCUSSION

Pain management has always been a very important aspect of breast augmentation surgery, which largely determines the length of recovery. Some people who want to have this surgery do not have it just because they feel that the pain after the surgery is severe.^6,8,11^ The degree of pain is closely related to the plane for implant placement.^10,19^ The traditional axillary approach with the implants beneath the pectoral major muscle is the most painful because of the large subpectoral dissection area and the high tension of the muscle due to implant insertion. In fact, the pain is mainly due to the implant under the intact pectoral muscle resulting in a significant increase in muscle tension, which is called “tension pain.”^10,11^ Tebbetts’ dual-plane technique alleviates postoperative pain because it partially releases the tension on the pectoral muscle.^1,2^ However, the release of this tension is not complete. Most of the pectoral muscle is still held tight by the implant, and hence, patients have also complained about this pain.^7,12^ How to further alleviate postoperative pain is still a significant problem to solve.^6-12^ Compared with the other plane, this is the least degree of pain experienced after subglandular and subfascial implant placement, yet there are strict indications for these 2 planes.^3-5^ Generally, the pinch thickness should be ≥20 mm; otherwise, implant palpability (especially in the lower pole) and ripples are common complaints.^5^ By combining the subfascial and submuscular planes, our modified technique completely released the tension of the pectoral muscle with the least amount of submuscular dissection. A part of the elevated pectoral muscle is completely separated from the part left in situ, and the tension release is complete; thus, the degree of postoperative pain is significantly reduced and BAD is eliminated.

Some surgeons also believe that after subfascial or subglandular plane surgery, mastoptosis may increase, the areola may enlarge, and the incidence of implant palpability may increase due to the lack of support by the pectoral muscle.^19,20^ Our procedure had the lower pole of the implant (which accounts for ∼30% of the implant's height) subpectoral and beneath the fasciae of the external oblique and the anterior serratus. This reverse dual plane ensures the thickness and supports the strength of the soft tissue that covers the lower pole of the implants.^21,22^ Some adverse consequences of subfascial or subglandular implant placement may decrease, such as increased mastoptosis, enlargement of the areolas, palpability of the implants, and capsular contracture.^19-22^

After surgery, most patients are satisfied with the softness of their breasts in the sitting position. However, when they lie in the supine position, their breasts usually become harder. As a result, some patients complain about the adverse effects on the quality of their sex life, which is at variance with the very purpose of the BREAST-Q.^13-16^ This result was confirmed in the last 6 years of follow-up with our patients. It is likely that most patients have not had to pay minute attention to their breasts, or that most surgeons have ignored it, and thus, we have not followed the literature in this regard to date. In a word, this phenomenon is an objective existence, we should come up with solutions.^14,15^

Anatomically, the stiffening of the breasts in the supine position, especially with the arms abducted, is mainly caused by increased pectoral muscle tension, as if the implants are oppressed by a thickened capsule.^21-23^ To solve this problem, implant placement plane and muscle tension release remain important. Our modified procedure made the upper and lower parts of the pectoral muscle to separate completely, with the upper part of the pectoral muscle being in situ. The tension of the pectoral muscle was completely released and the compression on the implants relieved. In Group A, only 50% of the patients felt no significant difference in the softness of their breasts between the supine and the seated positions. This meant that 50% of the patients felt that their breasts became harder in the supine position. In Group B, only 23.8% of the patients felt so, whereas the rest (76.2%) felt no significant difference in the different positions.

When performing this procedure, it is necessary to pay attention to the following details. First, when dissecting to the fifth rib, it must be ensured that the pectoralis major muscle is not cut perpendicular to the muscle surface, but rather is cut into a bevel from shallow to deep, so that the step deformity in the lower pole of the breast can be avoided after surgery. Second, attention should be paid to reserve the superficial branch of the intercostal nerve (especially the fourth intercostal nerve) during the dissection near the anterior axillary line and to protect the anterior recurrent branch of the intercostal nerve during the dissection near the parasternal line. Our method is to change sharp dissection to blunt dissection when it is close to the nerve. It should be added that this modified method is also feasible and applicable to both areolar and IMF incisions. We have performed this modified method on many patients through areolar and IMF incisions and will continue to share it in the future.

Finally, this study showed that our modification procedure significantly reduced postoperative pain, improved breast softness in the supine position, and rendered patients free from BAD. Our study had also some limitations such as the lack of quantitative evaluations of breast softness; thus, our modified procedure needs to be performed more frequently.

## CONCLUSIONS

Compared with Tebbetts’ dual-plane technique, our modified technique has a significant effect on relieving postoperative pain and improving breast softness in the supine position. In addition, it does not result in animation deformity and does not lead to an increase in other complications. The indications of the procedure and the need to evaluate breast softness quantitatively remain important and require further analysis.

## Supplementary Material

ojae020_Supplementary_Data

## References

[1] Tebbetts JB. Dual plane breast augmentation: optimizing implant-soft-tissue relationships in a wide range of breast types. Plast Reconstr Surg. 2006;118(Suppl 7):81S–102S. doi: 10.1097/00006534-200104150-00027 17099485

[2] Tebbetts JB. Axillary endoscopic breast augmentation: processes derived from a 28-year experience to optimize outcomes. Plast Reconstr Surg. 2006;118(Suppl 7):53s–80s. doi: 10.1097/01.prs.0000247314.92351.99 17099484

[3] Graf RM, Bernardes A, Rippel R, et al Subfascial breast implant: a new procedure. Plast Reconstr Surg. 2003;111(2):904–908. doi: 10.1097/01.PRS.0000041601.59651.15 12560720

[4] Brown T. A comprehensive outcome review of subfascial breast augmentation over a 10-year period. Plast Reconstr Surg. 2020;146(6):1249–1257. doi: 10.1097/PRS.0000000000007333 33234953

[5] Gould DJ, Shauly O, Ohanissian L, Stevens WG. Subfascial breast augmentation: a systematic review and meta-analysis of capsular contracture. Aesthet Surg J Open Forum. 2020;2(1):ojaa006. doi: 10.1093/asjof/ojaa006 33791626 PMC7671235

[6] Von Sperling ML, Høimyr H, Finnerup K, et al Persistent pain and sensory changes following cosmetic breast augmentation. Eur J Pain. 2011;15(3):328–332. doi: 10.1016/j.ejpain.2010.07.004 20727797

[7] Tan P, Martin M, Shank N, et al A comparison of 4 analgesic regimens for acute postoperative pain control in breast augmentation patients. Ann Plast Surg. 2017;78(Suppl 6):S299–S304. doi: 10.1097/SAP.0000000000001132 28459704 PMC6686898

[8] Govrin-Yehudain O, Matanis Y, Govrin-Yehudain J. Reduced pain and accelerated recovery following primary breast augmentation with lightweight breast implants. Aesthet Surg J. 2018;38(10):1092–1096. doi: 10.1093/asj/sjy071 29579148 PMC6137425

[9] Kang CM, Kim WJ, Yoon SH, et al Postoperative pain control by intercostal nerve block after augmentation mammoplasty. Aesthetic Plast Surg. 2017;41(5):1031–1036. doi: 10.1007/s00266-017-0802-6 28791441 PMC5605585

[10] Fredman R, Wu C, Rapolti M, et al Prepectoral direct-to-implant breast reconstruction: early outcomes and analysis of postoperative pain. Aesthet Surg J Open Forum. 2019;1(1):ojz006. doi: 10.1093/asjof/ojz006 33791602 PMC7984832

[11] Ciftci B, Ekinci M, Celik EC, et al Ultrasound-guided pectoral nerve block for pain control after breast augmentation: a randomized clinical study. Braz J Anesthesiol. 2021;71(1):44–49. doi: 10.1016/j.bjane.2020.12.004 33712252 PMC9373212

[12] Sforza M, Saghir R, Saghir N, et al Assessing the efficacy of the S-PECS block in breast augmentation surgery: a randomized, double-blind, controlled trial. Plast Reconstr Surg. 2024;153(1):1e–9e. doi: 10.1097/PRS.0000000000010492 37010475

[13] Leite AT, Sabino-Neto M, Resende VCL, et al Patient-reported outcomes after subpectoral breast augmentation with microtextured or macrotextured implants using the BREAST-Q. Arch Plast Surg. 2022;49(3):352–359. doi: 10.1055/s-0042-1748649 35832157 PMC9142250

[14] Mundy LR, Homa K, Klassen AF, et al Normative data for interpreting the BREAST-Q: augmentation. Plast Reconstr Surg. 2017;139(4):846–853. doi: 10.1097/PRS.0000000000003186 28350657 PMC5373485

[15] Arora N, Patel R, Sohi G, et al A scoping review of the application of BREAST-Q in surgical research. JPRAS Open. 2023;37:9–23. doi: 10.1016/j.jpra.2023.04.005 37288429 PMC10242639

[16] Xiong J, Hou Q, Hu Z, et al The application of anatomy combined with ultrasound knife in transaxillary endoscopic biplane breast augmentation. Front Surg. 2022;9:865379. doi: 10.3389/fsurg.2022.865379 35574545 PMC9091814

[17] Kim MK, Lee S. LigaSure for the creation of bloodless breast pockets in patients undergoing transaxillary breast augmentation. Plast Reconstr Surg Glob Open. 2020;8(12):e3295. doi: 10.1097/GOX.0000000000003295 33425607 PMC7787310

[18] Xue Y, Pu LLQ. Contemporary breast augmentation practice in the United States. Ann Plast Surg. 2021;86(3S Suppl 2):S177–S183. doi: 10.1097/SAP.0000000000002646 33346541

[19] Pereira LH, Sterodimas A. Transaxillary breast augmentation: a prospective comparison of subglandular, subfascial, and submuscular implant insertion. Aesthet Plast Surg. 2009;33(5):752–759. doi: 10.1007/s00266-009-9389-x 19597863

[20] Hauch AT, Francis CS, Artz JD, Chasan PE. Subpectoral implant repositioning with partial capsule preservation: treating the long-term complications of subglandular breast augmentation. Aesthet Surg J Open Forum. 2021;3(2):ojab009. doi: 10.1093/asjof/ojab009 34212143 PMC8240740

[21] Karabeg R, Jakirlic M, Karabeg A, et al The new method of pocket forming for breast implant placement in augmentation mammaplasty: dual plane subfascial. Med Arch. 2019;73(3):178–182. doi: 10.5455/medarh.2019.73.178-182 31404122 PMC6643325

[22] Hwang DY, Park SH, Kim SW. A modified dual-plane technique using the serratus anterior fascia in primary breast augmentation. Plast Reconstr Surg Glob Open. 2017;5(2):e1213. doi: 10.1097/GOX.0000000000001213 28280660 PMC5340475

[23] Mehra G, Kaufman-Goldberg T, Meshulam-Derazon S, et al Use of the subfascial plane for gender-affirming breast augmentation: a case series. Plast Reconstr Surg Glob Open. 2021;9(1):e3362. doi: 10.1097/GOX.0000000000003362 33564588 PMC7858195

